# Approaches to Formaldehyde Measurement: From Liquid Biological Samples to Cells and Organisms

**DOI:** 10.3390/ijms23126642

**Published:** 2022-06-14

**Authors:** Fedor A. Lipskerov, Ekaterina V. Sheshukova, Tatiana V. Komarova

**Affiliations:** 1 Vavilov Institute of General Genetics Russian Academy of Sciences, 119991 Moscow, Russia; fedor@lipskerov.ru (F.A.L.); sheshukova@vigg.ru (E.V.S.); 2 Chemistry Department, Lomonosov Moscow State University, 119991 Moscow, Russia; 3 Belozersky Institute of Physico-Chemical Biology, Lomonosov Moscow State University, 119991 Moscow, Russia

**Keywords:** formaldehyde, aldehyde derivatization, fluorescent probes, aza-Cope rearrangement, formaldehyde sensor

## Abstract

Formaldehyde (FA) is the simplest aldehyde present both in the environment and in living organisms. FA is an extremely reactive compound capable of protein crosslinking and DNA damage. For a long time, FA was considered a “biochemical waste” and a by-product of normal cellular metabolism, but in recent decades the picture has changed. As a result, the need arose for novel instruments and approaches to monitor and measure not only environmental FA in water, cosmetics, and household products, but also in food, beverages and biological samples including cells and even organisms. Despite numerous protocols being developed for in vitro and in cellulo FA assessment, many of them have remained at the “proof-of-concept” stage. We analyze the suitability of different methods developed for non-biological objects, and present an overview of the recently developed approaches, including chemically-synthesized probes and genetically encoded FA-sensors for in cellulo and in vivo FA monitoring. We also discuss the prospects of classical methods such as chromatography and spectrophotometry, and how they have been adapted in response to the demand for precise, selective and highly sensitive evaluation of FA concentration fluctuations in biological samples. The main objectives of this review is to summarize data on the main approaches for FA content measurement in liquid biological samples, pointing out the advantages and disadvantages of each method; to report the progress in development of novel molecules suitable for application in living systems; and, finally, to discuss genetically encoded FA-sensors based on existing natural biological FA-responsive elements.

## 1. Introduction

**Figure 1:**
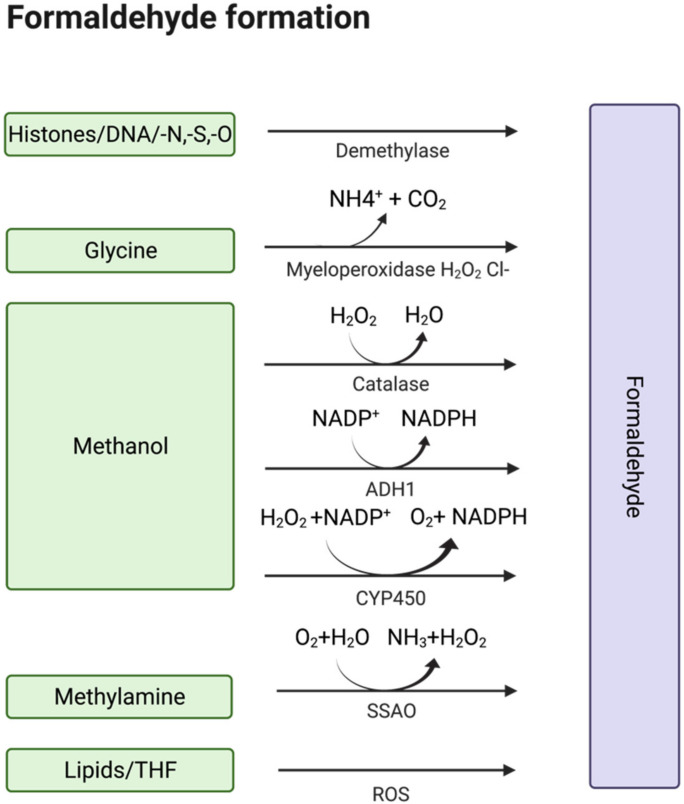
Sources of endogenous FA. Created with BioRender.com.

Formaldehyde (formula HCHO, abbreviated FA), is a simple, naturally occurring organic compound, is one of the first-row aldehydes and is the most poisonous of them [1,2]. It is a well-known fact that FA is a strong electrophile capable of reacting with various biological nucleophiles such as nucleic acids [3], and nucleophilic amino acids [4], thereby affecting the most important functions of living organisms. Nevertheless, FA is a normal component of the human organism itself, as physiological levels of FA are detected in exhaled air and blood. That fact became obvious with the development of methods by which FA could be assessed in biological samples [5]. Formaldehyde can enter the human body from outside—with polluted air or water, tobacco smoke [6,7], electronic cigarette vapour [8] and consumed food [9,10,11,12,13]. Moreover, there are numerous sources of endogenous FA as it is a (by-)product of different biochemical reactions in the organism performed by various enzyme systems, e.g., oxidative demethylation enzymes [14], semicarbazide-sensitive amine oxidases (SSAOs) [15,16,17], serine hydroxymethyltransferase [18], dimethylglycine dehydrogenase [19], lipid oxidation enzymes [20], P450 oxidase [21] and N-methyl group demethylase [22,23] (Figure 1). One of the main sources of FA in the human organism is exogenous and endogenous methanol that is oxidized by alcohol dehydrogenases, catalase and cytochrome P450 to FA [14]. Despite the continuous incoming flow of FA in the human organism in normal conditions, it is equal to the outgoing flow—FA concentration is maintained at low levels by different mechanisms of controlling its metabolism and clearance [14].

Nevertheless, in case of pathological conditions and malfunction of FA clearance systems, the increased levels of endogenous FA could lead to destructive and harmful processes. It can induce bone pain in cancer through TRPV1 activation [24] and it also plays a huge role in the origin and life of a cancer cell [25,26,27]. FA triggers cellular redox imbalance in human cells as it reacts with the redox-active thiol group of glutathione affecting the ratio of reduced glutathione and causing oxidative stress [28]. FA can bring genetic damage to hematopoietic stem cells, hepatocytes and nephrons [29]. Also, fluctuations of endogenous FA concentration are characteristic of the numerous pathological conditions associated with cognitive impairment [30].

FA could be assumed to be not only a by-product of endogenous biochemical processes but to also have some important functions. This assumption has risen into the hypothesis that human endogenous FA can act as a potential anticancer metabolite [27]. Moreover, endogenous FA was demonstrated to be a regulatory molecule that can help memory formation, particularly by enhancing NMDA (N-Methyl-D-aspartic acid) currents [31]. Recent studies have shown that FA can interfere with the activity of the glutamate receptor NMDA-R. FA binds one of the receptor subunits, thus endogenous FA enhances NMDA currents, while excess of FA suppresses NMDA currents by crosslinking amino acids of the NMDA-R subunit [32,33].

But how to measure FA concentration? The potential danger from exogenous FA was first discovered at the end of 19th–beginning of 20th century [34]. The first approach allowing FA concentration assessment was developed in the 1950s with the birth of gas chromatography. Methods for measuring FA were directed for its detection in waste water as well as in food and beverages [35], air and various types of alcoholic products [36]. For a long time, the scientific community did not consider the existence and significance of endogenous FA. But recent advances in molecular biology, biochemistry and physiology have revealed its importance indicating that FA can serve as a molecular marker for many normal and pathological biochemical processes in a living organism. It became necessary to optimize the existing FA detection methods to make them applicable for biological samples. According to that demand, numerous novel techniques were developed for FA content assessment in vitro, in cellulo and in vivo. Here we review a variety of approaches for FA measurement and monitoring that can be divided in to three main groups. The first group of methods includes gas and liquid chromatography and provide high sensitivity, selectivity, and reproducibility of measurements. However, these techniques are hardly applicable for FA assessment in real-time mode and in living cells and organisms. The second group comprises methods based on spectrophotometry detection of substances obtained in a reaction with FA. Some of these approaches could only be utilized for FA measurement in liquid samples (biological fluids, lysates, extracts etc.); but the others (mainly based on aza-Cope group rearrangement) could also be applied to monitor FA in cells and even living organisms. The third group includes genetically-encoded sensors that were developed for living cells and organisms. However, the variety of methods for FA assessment is not limited to those mentioned above: there are numerous other approaches for analysis of air samples using graphene- and metal oxide-based nanomaterial gas sensors [37,38,39,40,41], optical FA-sensors [42] and others that are out of the scope of this review.

The aim of this review is to highlight the latest developments in the field of FA detection in vitro and in vivo and their application in cellular and molecular biology, physiology and biochemistry, and to provide readers with a general overview of how FA could be assessed in biological samples. We summarize data on the approaches for FA content measurement in biological fluids as well as culture medium, cell extracts and lysates, and on the suitability of different methods developed for non-biological objects. We discuss the potential of the numerous probes applicable for real-time FA monitoring in cell culture, tissues and living organisms. And, finally, we focus on the recent advances in genetically-encoded FA-sensors.

## 2. In Vitro FA Measurement

The first objects in which it is necessary to measure the levels of endogenous FA are various biological fluids, such as blood, saliva, and urine as well as culture medium. Cell lysates and tissue extracts could also be assigned to this group of samples. As most of these samples contain proteins, the initial step of the sample preparation for chromatography is deproteinization that is usually performed by protein precipitation using trichloroacetic acid or acetonitrile. Measurement of FA in liquid samples does not need the invention of completely new methods, but rather, requires the use of known methods that are modified to achieve the best results.

### 2.1. Gas Chromatography

In early 1952, Martin and James carried out a variant of gas distribution chromatography (GC). Since that time, gas chromatography became one of the fastest growing methods in analytical chemistry. GC has many advantages over other measurement methods. Due to its high separation power, GC has found wide application in the food industry [43,44] and medicine [45,46]. No other method allows analysis of water samples or multicomponent liquid systems with hundreds of components in such a short time. Comparative simplicity of equipment management and ease of use are among the most important properties of gas chromatography. Gas chromatographs are relatively affordable, reliable, and allow an automated analysis process.

High sensitivity of the chromatograph makes it possible to determine small amounts of organic compounds with great accuracy. Today, gas chromatography can reliably determine concentrations of 10^−8^–0^−9^ mg/mL [47]. The obtained data may be widened when GC is combined with various instrumental methods such as mass spectrometry (MS) and Fourier-transform infrared spectroscopy.

GC–MS has become one of the most successful combined methods. By superposition of GC and MS, we get a tool combining the ability of GC to separate closely related molecules with the ability of MS to use data to identify and quantify separated substances.

However, GC has its drawbacks, which can sometimes become critical during analysis. With GC, it is impossible to separate and analyze mixtures of non-volatile compounds, thermally unstable compounds or compounds dissociating in the analyzed solutions.

#### 2.1.1. FA Derivatives’ Detection with GC

**Figure 2:**
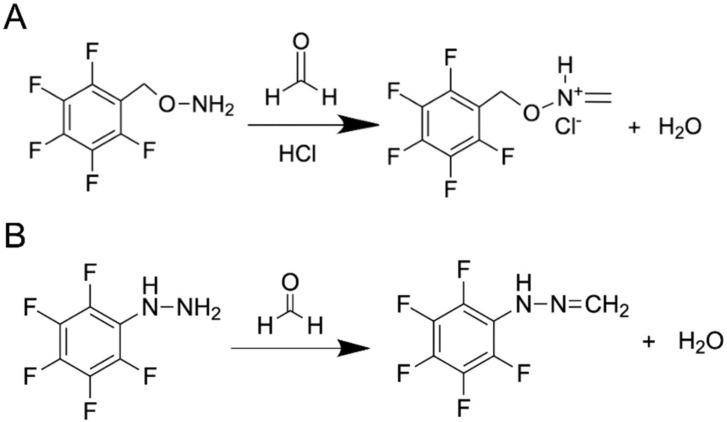
FA derivatization using PFBHA (O-(2,3,4,5,6-pentafluorobenzyl) hydroxylamine) (**A**) or PFPH (pentafluorophenylhydrazine) (**B**).

How can we use the GC method to detect such a small metabolite as FA with a mass of only 30.026 g mol^−1^? It is complicated and troublesome to catch this molecule without using special protocols of sample preparation. One of the common substances used for FA derivatization is O-(2,3,4,5,6-pentafluorobenzyl) hydroxylamine (PFBHA, CAS Registry No. 57981-02-9) (Figure 2A). Thus, FA derivatives–oximes–are obtained and then analyzed by GC coupled with flame ionization detector (FID), electron-capture detector (ECD) or mass spectrometry (MS). PFBHA has been known since 1975. It helps in detecting a variety of compounds among which are aldehydes [48,49]. PFBHA could be used for FA detection in water, blood [26], plasma, urine and amniotic fluid [50,51]. PFBHA reacts completely with FA within a few seconds, forming a GC-compatible oxime that is analyzed with a high-polarity capillary column and nitrogen as a carrier gas. The accuracy of FA detection using GC coupled with MS reaches the picogram (pg) level [52]. The PFBHA-based method has been recommended for FA detection by the US Environmental Protection Agency [53].

Another substance used for FA derivatization is pentafluorophenylhydrazine (PFPH) (Figure 2B). This approach was used to assess the FA content in biological samples such as homogenates obtained from different tissues [54], and the derivatization was performed directly in tissue homogenates in the presence of diluted (0.03–0.1 M) phosphoric acid (1–2 h 50–55 °C) followed by analysis of pentafluorophenyl hydrazone by GC–MS using a capillary high polarity column and helium as a carrier gas. This method allows the detection of FA at concentrations that are characteristic of natural endogenous levels for mammals [55]. However, PFPH is mostly used for FA detection in non-biological samples.

The products of FA derivatization are usually recovered using liquid–liquid [55] or liquid–solid phase extraction methods [56], but these procedures greatly complicate the analysis. Therefore solid-phase microextraction (SPME) is the most convenient approach [57,58]. There are several approaches based on the joint usage of extraction and derivatization: the reaction could be performed in the working solution using headspace or liquid SPME [59] as well as derivatization on fiber [60]. On the other hand, for on-fiber derivatization, a derivatizing agent could be applied to the fiber and then exposed to the headspace of the sample [52,61]. This allows FA to be measured accurately and to obtain reliable values every time.

#### 2.1.2. GC with Catalytic Hydrogenolysis Coupled with a Flame Ionization Detector (FID)

Although the GC analysis of FA derivatives detected with FID/ECD and especially MS described above provides high accuracy, it is costly and time-consuming. Jim Luong and colleagues developed a new GC technique: using a capillary column by which FA, acetaldehyde, and other components were separated from a gas-phase matrix, then the aldehydes were converted into methane and ethane, respectively, using a nickel-coated catalyst in a hydrogen atmosphere. The methane and ethane were then detected using an FID [62].

Further, the catalyst-based method was upgraded and catalytic hydrogenolysis was performed directly in a 3D-printed FID jet consisting of an FID combined with a catalyst support for hydrogenolysis. The sensitivity of this approach reaches ppm level in the range of 0.5–300 ppm [63]. The main advantages of this method are the absence of the need for preliminary preparation, derivatization, and concentration of the sample. This method can be used safely for the FA measurement in biological fluids after proper separation of the aqueous phase (precipitation of proteins, centrifugation). This promising approach has excellent prospects and the FID jet is already commercialized as Jetanizer^(TM)^ and available.

### 2.2. Liquid Chromatography

Liquid chromatography (LC) is a method for separation and analysis of complex mixtures of substances in which the mobile phase is liquid [64]. The mobile phase in LC performs a double function: (1) it provides the transfer of desorbed molecules along the column (as in the mobile phase in GC); (2) it regulates the equilibrium constants, and consequently the retention, as a result of interaction with the stationary phase (adsorbed on the surface) and with the molecules of the substances being separated. By combining various sorbents and a huge number of mobile phases different in their composition, it is possible to solve many tasks [65]. The LC method is applicable for the separation of a much wider range of substances than GC, since most of the substances are not volatile and are unstable at high temperatures. LC separation is usually performed at room temperature.

High performance LC (HPLC) can be applied to the analysis of various very small molecules [66] which are involved in biological processes in living organisms. HPLC has long been used to work with systems of biological origin, but FA cannot be measured directly by LC due to the chemical and physical properties of FA. Moreover, the concentrations of endogenous FA in biological samples are very low. Thus, it is necessary to obtain more stable and less reactive derivatives as the products of irreversible reactions with FA. These FA derivatives could be analyzed using HPLC combined with different detectors (usually UV) or MS.

#### 2.2.1. 2,4-Dinitrophenylhydrazine for FA Measurement

**Figure 3:**
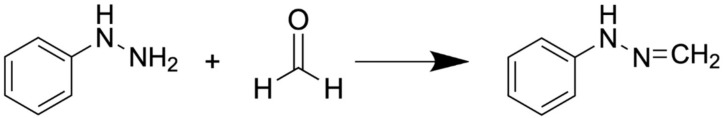
2,4-dinitrophenylhydrazine reaction with formaldehyde.

The most widely used substance for binding FA is 2,4-dinitrophenylhydrazine (DNPH) [67]. As a result of this reaction, 2,4-dinitrophenylhydrazone is formed (Figure 3). This compound is separated using reverse-phase HPLC with acetonitrile/water mobile phase and hydrophobic stationary phase (columns with C_18_ ligand bonded to silicon dioxide are commonly used), and monitored using a UV detector [68]. The FA derivative with DNPH is preferred for HPLC to any other reagents because it can be monitored in the region of 330–360 nm which is more specific compared, for example, with formaldemedone (see below) with detection at 254 nm. Moreover, this approach based on DNPH–HPLC allows the simultaneous determination of FA and methylglyoxal [69]. As in the case of GC, it is possible to increase the efficiency and accuracy of measurements by using a combined approach of MS and HPLC [70]. DNPH–HPLC can be applied to virtually any non-biological sample including water [71,72], drug substance [73], food [74], leather [75], etc. This method also allows the measurement of FA in biological samples like tissue homogenates [76], urine [69], blood and serum [77,78,79]. Despite the wide use of this approach, it still requires elaborate sample preparation and special equipment, which is not always accessible.

#### 2.2.2. Ampicillin-Based Reaction for FA Derivatization and HPLC Analysis with Fluorescence Detection

**Figure 4:**
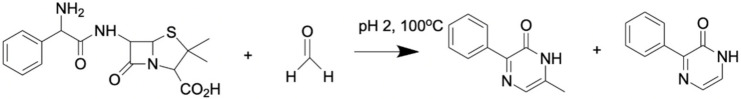
FA reaction with ampicillin results in formation of fluorescent pyrazine-2-one.

Another substance for FA derivatization is ampicillin (D-(2)-a-aminobenzylpenicillin) that reacts with FA forming a fluorescent product (Figure 4) [80] analyzed by reverse-phase HPLC (acetonitrile/water mobile phase and a column with C_18_ ligands) with fluorescence detection (excitation and emission wavelengths are 346 nm and 422 nm, respectively). This approach was first developed for ratiometric ampicillin detection in animal tissues [80], but later it was demonstrated to be applicable for FA measurement as well [67]. However, Reinbold and colleagues recently characterized the structure of the fluorescent product of ampicillin/FA reaction and concluded that this method of detection is not selective enough because the same or similar fluorescent compounds (pyrazinones) could be formed with other biologically relevant molecules [81]. This finding indicates that chromatographic separation of reaction products before detection is essential to distinguish between FA-derived and other similar pyrazinones, and proper controls should be set because ampicillin reaction with glyoxylate gives exactly the same adduct as with FA [81].

#### 2.2.3. Dimedone for FA Derivatization

**Figure 5:**
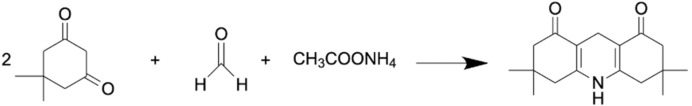
FA derivatization with dimedone.

E. Tyihák and colleagues used dimedone as a derivatization agent for FA [82]. This reaction (Figure 5) is similar to the Nash reaction [83] as it is also based on the condensation reaction between FA and a reagent consisting of β-diketone and ammonium acetate. A solution of dimedone in methanol was demonstrated as a suitable reagent for the isolation of FA from different biological samples. Moreover, methanol in this protocol plays the role both of extractant and eluent [82], and no additional deproteinization stage is needed due to simultaneous extraction and derivatization. The reaction product, formaldemethone (Figure 5), could then be analyzed by reverse-phase HPLC (methanol as a mobile phase and C_18_-ligand column) with UV detection (260 nm). Using this approach, FA assessment was performed in biological samples such as tissue (liver, muscle, tooth) lysate, urine and plant extract [82,84,85].

#### 2.2.4. Detection of Reaction Products of FA and Amino Acids

**Figure 6:**
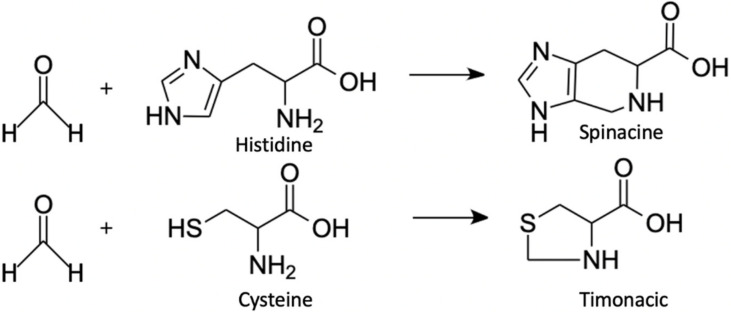
FA reaction with histidine and cysteine to form spinacine and timonacic respectively.

It is a well-known fact that FA reacts with different amino acids [86,87,88]. But until recently, this fact was considered only for exogenous FA, which negatively affected the organism. The elevated levels of FA/amino acid adducts are regarded as biomarkers of FA exposure [89,90]. The main focus has been on human serum albumin lysine [91] and human hemoglobin valine [92] adducts that could be detected and analyzed with LC-MS. However, besides monitoring the effects of exogenous FA, its adducts could give information on the levels of endogenous FA being some kind of a reservoir of FA. It has been shown that ADH5-deficient cells recycle FA via alternative pathways [93]. Moreover, FA applied to mammalian cell culture reacts with free cysteine and histidine residues, forming products that are stable over a wide pH range [88]. Such spontaneous reactions result in the formation of timonacic and spinacine (Figure 6), respectively [88]. To assess timonacic and spinacine concentrations, they are extracted from cells or serum with the mixture of acetonitrile–methanol–water (3:5:2) and then analyzed by HPLC–MS using a hydrophilic interaction chromatography (HILIC) column with a high-performance zwitterionic stationary phase attached to porous polymer beads [88]. The reaction product between cysteine and FA, timonacic, is more soluble than cysteine and less reactive than FA [93], which facilitates its transfer through tissues. Cysteine conversion to timonacic is reversible, which is why timonacic could be regarded as a kind of FA “storage”, which forms in some tissues and then enters the bloodstream. In the blood, it is converted back to cysteine and FA, thereby promoting the distribution of cysteine and FA throughout the body [93]. Due to all the properties described above, timonacic can be used to track relative changes in FA concentration in blood [88,93], but the absolute FA concentration cannot be assessed by this approach.

To conclude, HPLC and GC are precise, selective and well-established methods for the detection of FA in various samples, including biological. However, they are only suitable for in vitro detection. Moreover, additional steps of sample preparation, including deproteinization and derivatization, are necessary. Therefore, if all these criteria are applicable to the particular research task and the corresponding equipment as well as a qualified operator is available, then chromatography represents one of the best choices for FA assessment.

## 3. From In Vitro to In Cellulo

### 3.1. Chromogenic or Fluorogenic FA Chemosensors

There is a group of methods used for FA concentration assessment based on the measurement of absorbency or fluorescence of the products obtained in the FA reactions with particular substances [94,95]. Spectrophotometry allows measurement of chromogenic and fluorogenic substances, obtained as a result of such reactions. Most of these protocols are used for in vitro FA quantification as very convenient methods because they allow analysis of multiple samples in automatic mode (for example, microplate format); and only common devices found in molecular biology laboratories are needed including spectrophotometer, rather than specialist equipment such as HPLC or GC devices. As data on the role and function of endogenous FA in living systems accumulated, and more and more information appeared around the participation of this molecule in various processes associated with diseases, a need arose for relatively simple and widely available methods for measuring FA in biological samples in vivo in real-time conditions. The benefit afforded by the high sensitivity of the GC and HPLC methods is largely offset by the need for expensive specialist equipment and a qualified operator. Moreover, such approaches cannot be exploited for in vivo FA monitoring. By contrast, fluorescent microscopy and special FA-sensing probes allow monitoring of FA levels in cell cultures and even animals. The main criteria for the substance to be suitable for FA monitoring in living systems are low toxicity, stability, cell-penetration ability, and selectivity against other aldehydes that are present in the cell. For in vivo monitoring, a near-infrared excitation/emission profile is also preferable. Below, we discuss the application of FA-sensors for in vitro application, and adjustment of these molecules or development of completely new ones to make them applicable for in vivo context.

#### 3.1.1. β-Diketone Esters and Hantzsch Reaction

**Figure 7:**
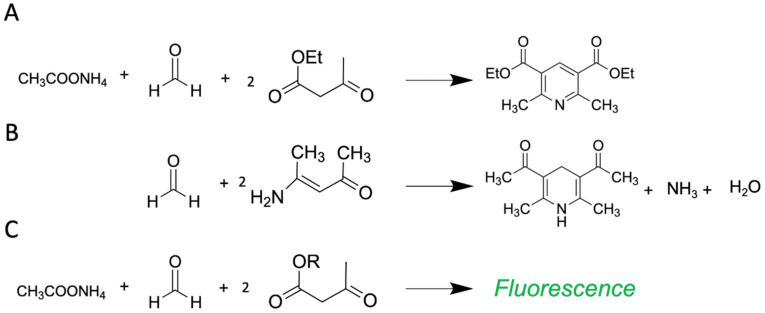
(**A**) Hantzsch reaction used for FA detection by Nash approach, (**B**) Fluoral-reaction with FA, and (**C**) polymer-based application of Hantzsch reaction. R denotes polymer.

This reaction was known back in 1881 thanks to Arthur Rudolf Hantzsch [96]. Subsequently, this reaction found its use for measuring FA in biological samples in 1953, when Nash modified the reaction and used acetylacetone [83] (Figure 7A). This allowed him to measure the FA content in suspensions of living bacteria for the first time. Since then, this colorimetric reaction could be carried out under milder conditions. The acetylacetone-based protocol is now mainly used for FA detection in non-biological samples such as textiles [97], cosmetics [98], beverages [99] etc. Nevertheless, a method is suitable for biological in vitro samples: FA content was assessed in rat hepatocyte lysates [100], human erythrocyte lysates [101], hemolysate [102] and mushroom extracts [103]. Moreover, a commercial kit for different biological samples was developed (Hach Company).

Nowadays, this reaction of β-diketone ester, an aldehyde and ammonia or an alkylamine is actively used for the spectrofluorometric determination of a wide range of non-fluorescent substances which contain a primary amino group, as well as for FA detection. Li and colleagues introduced a novel reagent for FA measurement based on Hantzsch reaction, namely, acetoacetanilide (AAA) [104,105]. The AAA reaction with FA is carried out at room temperature and its product can be detected by absorbency or fluorescence. The protocol was initially made for FA assessment in water but further developements based on this method resulted in the manufacture of commercial kits for FA measurement in biological samples with sensitivity 1.5 µM (Sigma-Aldrich, BioAssay Systems, Abcam) and successfully used for FA levels monitoring in cell and tissue lysates [33,106] and cerebrospinal fluid [107].

Another reagent for FA measurement, Fluoral-P (4-amino-3-penten-2-one), was developed [108]. Fluoral-P is colorless, while the product of reaction with FA, 3,5-diacetyl-1,4-dihydrolutidine (DDL), is yellow and can be detected by a spectrophotometer at 420 nm (Figure 7B). As many others, this protocol was initially applied to FA monitoring in air samples [109,110]. Subsequently, this approach was used by Yue and colleagues to estimate FA content in brain tissue lysates [111]. The method showed high sensitivity (LOD for detecting FA was 0.5 μM) and was demonstrated to be applicable for biological samples—in this case, brain tissue lysates [111].

Due to biotoxicity, and the instability of some of the components and products of the Hantzsch reaction, the latter was limited to the detection of endogenous FA in vivo. However, a new approach based on polymer chemistry has given the Hantzsch reaction a second wind allowing in vivo application. Instead of small molecules as FA-sensors (e.g., acetylacetone), different polymers containing the corresponding functional group (β-diketone ester) could be used for FA detection via the Hantzsch reaction (Figure 7C). The application of this approach has yielded remarkable results. The FA-sensor-supplemented polymer structures are highly biocompatible [112,113]. Thus, it has been shown that these polymers can efficiently detect endogenous FA in cell culture or in living organisms (e.g., zebrafish). Liu et al. [114] recently reviewed in detail how to use polymer chemistry to create a suitable FA measurement model, and Pan et al. [113] summarized data on the progress in polymer-based FA-sensors and discussed the advantages of polymeric probes for FA detection in vitro and in vivo. The polymer-based approach opens a new horizon for bioimaging and expands the field of application of the Hantzsch reaction.

#### 3.1.2. Fluorescent FA-Sensors Based on Formimine, Hydrazine and 2-Aza-Cope Reactions

Recentl excellent reviews by Xu et al. [115], Manna et al. [95] and Pan et al. [113] summarized advances on fluorescent probes and sensors, which were developed in the last decade and can be used to monitor FA in a variety of objects both in vitro and in vivo. Most of the substances described in these reviews are suitable for living cells and were demonstrated to be successfully applied for FA level assessment. We, in turn, briefly mention here the main types of reactions with several examples. The main reactions which are the basis for numerous FA-sensing probes for in vivo application are: formimine, hydrazine and 2-aza-Cope reactions.

**Figure 8:**
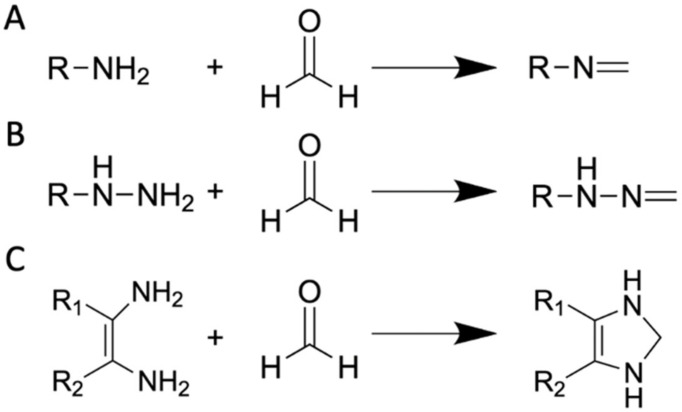
Schematic representation of the reactions for FA detection based on (**A**) formimine-, (**B**) hydrazine- and (**C**) diamine-containing probes. R denotes fluorophore-containing moiety.

- Methods based on formimine and aminal-moiety reactions (Figure 8).

The probes were developed based on difluoro boron dipyrromethene (BODIPY) fluorophore and a primary amino group as a FA-responsive element. The prototype probe, aniline-substituted BODIPY (AnB), was suggested by Song et al. in 2012 [116]. Later, they created another variant—BOD-NH2—that was shown to reversibly react with FA and be suitable for FA monitoring in peritoneal cavity of mice in vivo and assess FA concentration ex vivo in different mice organs [117]. Another probe—BODIPY-OPDA—based on a similar principle was developed by Cao et al. [118]. Both probes were demonstrated to penetrate to the cultured mammalian cells and allowed the detection of FA fluctuations [117,118].

Several probes based on rhodamine fluorophore variants supplemented with different diamine moieties were developed: dRB-EDA [119], R6-FA [120]; ortho-diaminorhodamine [121]. These probes showed high sensitivity in vitro (<10 µM) and were demonstrated to have negligible cytotoxicity so they were suitable for FA monitoring in living cells [120,121]. However, both probes appeared to be applicable only as a tool for visualization rather than quantitative measurement in cellulo.

Chen and colleagues developed a ‘turn-on’ probe based on tetraphenylethene functionalized with two amine groups [122]. The reaction with FA leads to the change in solubility of the resulting product making it insoluble and fluorescent. The probe makes it possible to detect very low FA concentrations (<1 µM) and is suitable for endogenous FA monitoring in cells.

- FA-sensors based on hydrazine mechanism

The probes based on the hydrazine mechanism (Figure 8) usually contain fluorophore (1,8-naphthalimide or BODIPY, for example) coupled with hydrazine as the FA-reactive moiety. In view of in vitro applications, most of the developed sensors have low cytotoxicity and allow the qualitative detection of FA in living systems. The sensitivity and selectivity of these probes is high enough to visualize both exogenous and endogenous levels of FA in living cells. Below we discuss examples and variations.

Chen and colleagues developed a probe based on hydrazine-substituted BODIPY [123]. This FA-sensor is colorless and not fluorescent in the PBS, but in presence of FA a hydrazone moiety is formed resulting in fluorescence and solution color change. The method is very sensitive (LOD is <1 µM) and highly selective over other aldehydes (glyoxal, methylglyoxal, acetaldehyde). Moreover, it has low cytotoxicity. All this makes it a very promising tool for in vivo qualitative detection of endogenous cellular FA.

Another ‘turn-on’ FA-probe—NA-FA—previously developed by Dr. Lin’s group was based on 1,8-naphthalimide as a fluorescent chromophore and hydrazine as an interaction site with FA [124,125]. This NA-FA probe was shown to have very low cellular toxicity thus to being suitable for in vivo FA monitoring in cells. Furthermore, the NA-FA was upgraded and several organelle-specific FA-sensors were developed in Dr. Lin’s laboratory: (1) lysosome-targeted Na-FA-Lyso [126] where morpholine was used for specific delivery to the lysosome; (2) endoplasmic reticulum-targeted NA-FA-ER probe was created by addition of methyl sulfonamide moiety to NA-FA for ER targeting and FA visualization in the ER [127]; and (3) NA-FA-MT probe containing triphenylphosphine as a mitochondria-targeting signal [128]. All these probes were demonstrated to have high signal-to-noise ratios, low detection limit (<1 μM) and high selectivity. Also this research group developed a biotin-containing FA-probe that is specific to cancer cells that over-express biotin-selective transporters [124]. This probe enables the observing of an increase in the exogenous and endogenous FA content in cancer cells and can find application as a tool for assessment of endogenous FA levels in cancer tumors. Presumably more generally it may be used in inflammatory processes associated with the appearance of FA.

We believe that in time, approaches will be developed based on the organelle-specific probes that will allow for quantitative detection of FA directly in the organelle of interest, and a more precise study of various processes associated with the appearance of FA will thus be possible.

- 2-aza-Cope probes for FA detection

2-aza-Cope chemistry requires special attention, as this approach can be used to quantitatively measure FA in living objects [129]. FA can enter into a 2-aza-Cope regrouping, a quick rearrangement with a low activation barrier [130]. This method of measuring FA differs from all others by the mechanism of reaction.

**Figure 9:**
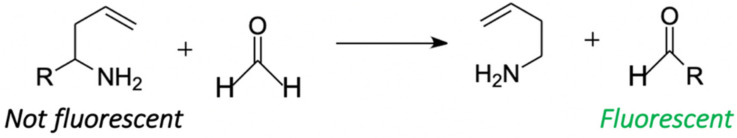
FA in 2-aza-Cope regrouping. R denotes probes for FA detection.

The approach based on this rearrangement gave a basis for multiple tools for a qualitative and quantitative measurement of FA, which are not affected by the illumination of the sample and the change in the localization of the chromophore in living objects [131]. Christopher Chang’s laboratory [132,133] and Jefferson Chan’s laboratory [134] are at the forefront of selective molecular imaging. Chang’s group has developed a variety of activity-based sensing methods based on the 2-aza-Cope reaction (Figure 9) for measuring and visualizing FA in living objects [133]. By now, more than 10 probes for the detection of FA in different systems have been developed, synthesized, and investigated by this research group. And in general more than 30 probes have been successfully tested as FA-sensors in living systems [129]. It was shown that 2-aza-Cope rearrangement-based probes are a universal tool and can be applied to a wide variety of imaging platforms, such as multicolor and ratiometric fluorescence [135], positron emission tomography detection [136] and chemiluminescence [131]. This allows for real-time monitoring of endogenous FA levels in mammalian cells and in vivo in laboratory animals [129,136]. One of the main drawbacks of the 2-aza-Cope rearrangement-based probes is slow reaction kinetics: for first generation probes, the reaction took from 1 to 2 h [26] but later, with the invention of novel substances, it was shortened to 30 min [135]; and recently, the FormAFP probe was developed that, within minutes, provides rapid, selective and sensitive (LOD 66 nM) FA detection in cultured cells [137].

With the use of unique structures capable of entering 2-aza-Cope regrouping and the high reactivity of these analytes to FA, it is possible to work with a large number of different model organisms.

Further development of 2-aza-Cope reactions may in the future offer new approaches to the study of the role of FA in biological systems and even in humans.

## 4. Genetically-Encoded FA Biosensors

Recently, novel approaches based on the genetically encoded biosensors were developed. The main advantages of such sensors are: FA levels assessment in vivo; the convenient methods of detection based on the fluorescence measurement; and no dependence on expensive equipment. The invention of the first genetically-encoded FA biosensor was based on the study of pathways for FA detoxification in bacteria. To-date, only few genetically-encoded FA biosensors applicable for bacterial cells have been developed [138,139,140], and until recently there was no biosensor adapted for eukaryotic cells. However, based on the properties of the *Bacillus subtilis* HxlR transcription factor sensitive to FA [138,141], Zhu and colleagues developed a FA biosensor applicable for mammalian cells [142]. This invention opens up new opportunities for real-time in vivo monitoring of FA in cells and tissues.

### 4.1. Escherichia coli Frm Operon and FrmR Transcription Factor

**Figure 10:**
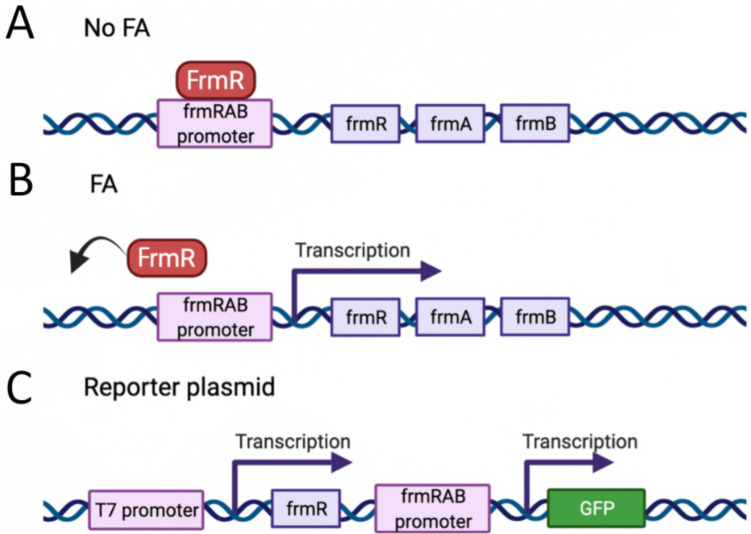
(**A**) FrmR repressor protein binds to the promoter region, preventing transcription of the downstream genes. (**B**) When FA concentration increases, it reacts with the nucleophilic Cys36 of FrmR, inducing its conformational changes; this leads to FrmR dissociation from the promoter, and makes the promoter available for DNA-dependent RNA polymerase. (**C**) Schematic representation of the reporter plasmid encoding GFP under control of frmRAB promoter. Created with BioRender.com.

The *E. coli* FA detoxification system contains a protein FrmR [141,143,144] which regulates the frmA/frmB operon encoding glutathione-dependent formaldehyde dehydrogenase gene [145] and S-formylglutathione hydrolase gene [146], respectively. FrmR is encoded by the same operon (Figure 10). Tralau and colleagues developed a FA biosensor system based on FrmR properties [139]. They obtained a plasmid containing a frm operon fused to the GFP reporter gene. In absence of FA, the FrmR repressor protein binds to the frmRAB promoter preventing GFP transcription. FA reacts with the nucleophilic FrmR Cys36 and induces a conformational change that leads to FrmR dissociation from the promoter (Figure 10) resulting in GFP expression. FrmR is specific to FA, as it responds to a far lesser degree to acetaldehyde, methylglyoxal or glyoxal, and not at all to a range of other aldehydes and alcohols tested [143,144,147]. Later, Rohlhill and colleagues performed screening of the FrmR promoter to reveal the principal nucleotides for its functioning, and obtained substituted variants that demonstrated up to 14-fold lower basal expression, 13-fold higher induced expression, and a 3.6-fold stronger response to FA in a prokaryotic system [147]. Subsequently, the development of genetically encoded biosensors for monitoring the concentration of FA in prokaryotic systems began to be seen. Further, Woolston and colleagues optimized the native FrmR binding site and obtained a much more sensitive biosensor enabling FA detection at levels as low as 1 μM [140], an LOD close to the Nash application of the Hantzsch reaction [83]. This approach was further utilized in prokaryotic cells to measure in vivo activity of several variants of NAD-dependent methanol dehydrogenase (Mdh) [140]. A similar design was used for the development of the new genetic enzyme screening systems (GESSs) to detect formate, FA, and methanol from specific enzyme activities and pathways [148].

### 4.2. Bacillus Subtilis HxlR Transcription Factor

One of the transcription factors regulating the FA detoxification pathway in bacteria, *B. subtilis* HxlR1, was similarly used to create a novel FA-sensor [138,141]. J. Law predicted Cys and Lys residues to be responsible for HxlR FA sensing [141], and later Zhu and colleagues crystallized FA-activated HxlR/DNA complex confirming this prediction [142]. It was demonstrated that FA induced an intrahelical crosslinking reaction between the side chains of Cys11 and Lys13 residues on the HxlRα1 helix, leading HxlR to undertake an optimal conformation for DNA binding.

**Figure 11:**
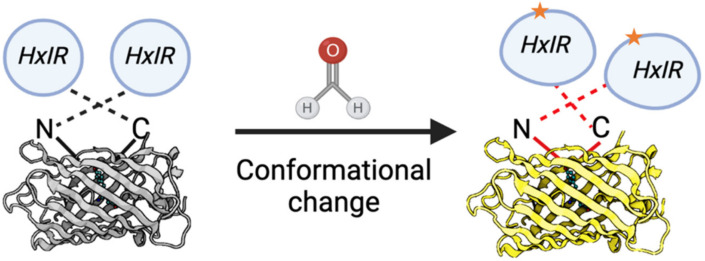
Schematic representation of the FA-sensor FAsor that contains cpYFP flanked with two copies of HxlR. FA induces “N-terminal helix-flipping” in each HxlR unit linking Cys11 and Lys13 residues on the HxlRα1 helix (crosslinked residues are marked with an asterisk). This event leads to a major conformational change in cpYFP module resulting in an increase in fluorescence.

Zhu, R. and colleagues developed a first FA-sensor (FAsor) for FA detection in vitro and in vivo for eukaryotic cells based on the following systems: (1) circularly permuted fluorescence proteins (cpFPs) the ability of which to emit fluorescence is highly sensitive and strongly depends on their conformation; and (2) the ability of HxlR to undergo conformational changes induced by FA [142]. By fusing cpYFP with two HxlR subunits, they obtained a sensor HxlR-cpYFP-HxlR designated FAsor that can translate FA-induced conformational changes into a change in fluorescence signal (Figure 11). This sensor could successfully perform in buffers with pH between 6.6 and 8.2 and is sensitive enough to emit detectable fluorescent signal in solution containing as little as 20 μM FA, thus making it possible to measure the physiological concentrations of FA and their fluctuations [142]. FAsor was also shown to be effective in transfected living cells and tissues. This approach allows different subcellular targeting of the sensor using signal sequences. All FAsor variants with organelle-specific signals were demonstrated to have the desired subcellular localization: nucleus, cytoplasm, and mitochondria. At the moment there is only one significant limitation of this system: FAsor is sensitive to another endogenous aldehyde, namely methylglyoxal [142]. Nevertheless, in view of the opportunities that have been opened by the development of this sensor [142,149], the above-mentioned feature does not impose a significant restriction, since the level of methylglyoxal in living cells is much lower than FA.

### 4.3. Methylorubrum Extorquens EfgA-Based Sensor

With their great diversity of different metabolic pathways, bacteria appear to be a golden mine that could give us a variety of FA-sensors. However, besides the above-mentioned Frm and Hxl that are already exploited in the development of FA-detecting systems, only one more FA-sensitive mechanism in a bacterial cell was recently discovered [150]. This sensor turned out to be the EfgA protein from *M. extorquens* PA1 [151]. This protein could bind FA and induce translation arrest to prevent protein damage in response to elevated levels of endogenous FA. Thus, EfgA is a unique mechanism of the stress response in bacteria to elevated levels of a toxic intracellular metabolite [150]. The ability to function in the same way in heterologous bacteria *E. coli* indicates the universal nature of this mechanism. The development of an FA-sensor based on EfgA properties might be not as appealing as for Frm and Hxl. Its potential sensitivity seems to be much lower as the background intracellular FA concentration during the steady-state growth of *M. extorquens* on methanol has been estimated to be 1 mM. Nevertheless, any new FA-sensitive system for in vivo use could widen the range of instruments for FA monitoring and measurements.

### 4.4. Combining Genetically Encoded Sensor with 2-Aza-Cope Reaction

Two genetically encoded FA probes were recently developed [152]. The approach used is based on the incorporation of an unnatural amino acid (UAA) [153] residue into the reporter protein. Zhang and colleagues designed and synthesized a lysine-derived UAA that contains the 2-aza-Cope reactivity and could interact with FA, and designated it PrAK. This UAA replaces lysine residue (K85) in GFP, preventing the protein from proper folding and almost incapacitating it for fluorescence. Firefly luciferase (fLuc) reporter protein also contains a lysine residue in 529 position that is essential for fLuc enzymatic activity. When FA concentration in the medium or in the cell increases, the UAA reacts with FA followed by a 2-aza-Cope rearrangement. The result is that essential lysine residue is “repaired” and the reporter protein recovers its functional activity: GFP becomes fluorescent, while fLuc restores enzymatic activity [152]. The developed approach was confirmed to provide enough sensitivity in vitro and in vivo in bacterial and mammalian cells to detect FA in a concentration-dependent manner in the range of 0.1–1 mM. Moreover, the GFP-based system is applicable to monitor the levels of endogenous FA in response to THF (2 mM) or 5,10-me-THF (1 mM). Despite the obvious prospects of exploitation of such genetically encoded sensors to assess FA levels in vivo, there are numerous challenges to be considered, however. The whole system is based on the usage of in-house synthesized PrAK and the stably-transformed cell line that contains modified pyrrolysyl-tRNA synthetase able to operate with PrAK. These features might restrict the availability and applicability of this approach and prevent its wide use at present.

## 5. Conclusions

**Figure 12:**
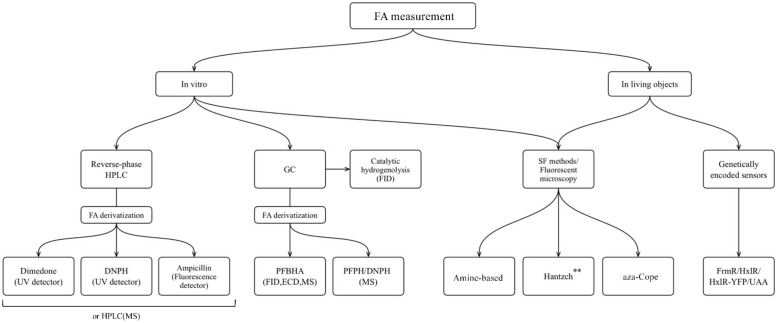
The approaches for FA measurement applicable for different biological samples: HPLC, high performance liquid chromatography; GC, gas chromatography; MS, mass spectrometry; FID, flame ionization detector; ECD, electron-capture detector; SF, spectrophotometry. ** Only Hantzch polymers that do not show cytotoxicity could be used for living objects.

FA is one of the products of cellular metabolism, and monitoring of FA levels can give us information about physiological and pathological changes in living systems. The aim of this review was to present the main categories of methods that have been optimized and applied to FA measurement in liquid biological samples, as well as to demonstrate the progress in development of novel molecules suitable for application in living systems. Finally, an additional aim was to discuss genetically encoded FA-sensors based both on existing natural FA-responsive elements, and on the combination of aza-Cope chemistry and induction of protein conformational changes. We presented an overview of the methods that are widely used for FA measurement in non-biological samples and are based on FA chemical reactivity; and we assessed challenges that occurred in their transition from non-biological to biological samples. We summarize the approaches discussed in the flowchart below (Figure 12).

For detection of FA levels, characteristics of biological objects require the use of methods with high sensitivity, since endogenous FA concentrations and their fluctuations are in a micromolar range. In addition, these methods must meet several criteria: selectivity, reproducibility and resistance to interfering substances that may present in biological samples. Often, the amount of biological samples is limited, so this parameter should also be taken into account. The most precise and selective tools for the assessment of FA concentrations in liquid biological samples are GC-–MS and HPLC–MS. However, their main disadvantages are the elaborate equipment required, and the complexity of the procedure and data interpretation. The alternative to chromatography is to use spectrophotometry-based methods. They are sensitive enough for detection of endogenous levels of FA, selective, easy to operate, suitable for microplate format and commercially available.

When it comes to measuring FA in living systems, additional requirements are added: low or negligible cytotoxicity and absence of side-effects on biological processes in cells are the essential features of such probes for monitoring FA in experimental animal organisms. Moreover, the probe emission should be close to the near-infrared range. The substance used for FA detection must be stable in the culture medium, inside the cell, and in the organism. Most probes for in vivo FA monitoring work on the basis of the aza-Cope rearrangement mechanism. Previously, the disadvantage of such substances was slow and irreversible reactions, but recently-developed novel molecules react rapidly thereby overcoming this drawback. The fundamental challenges in the innovative field of in vivo FA measurement relate to the lack of control over the concentration of the probes in a living organism due to physiological and biochemical processes. It is therefore necessary to assess the potential generalized effects of each novel probe and its “behavior” in a living system as this could complicate its usage in the research of the FA role in the organism. Currently, only short-time in vivo FA monitoring can be performed in a living organism because of the decrease of the probe concentration and its half-life.

Progress in the development of new probes for FA measurement in living systems promise to result in obtaining a new universal sensor, devoid of these drawbacks and meeting the required criteria in the near future. However, most of FA-sensing molecules developed for living systems are substances synthesized in-house and so are not available for wide use which impedes the application of this valuable tool for research.

Genetically-encoded FA-sensors have great potential as they are sensitive enough for endogenous FA level detection, could be targeted to different cellular compartments, have no cytotoxicity and do not require addition of chemical compounds to the cultured cells. However, one of the drawbacks is their sensitivity to methylglyoxal. Moreover, in vivo application of such sensors is not available yet. Thus, future efforts in upgrading of genetically-encoded sensors should include adjusted selectivity to FA and suitability for in vivo application.

A new generation of FA-sensing tools suitable for living objects is expected to be developed to meet the requirements mentioned above. The commercialization of novel probes thus making them available to the research community at large would greatly enhance research potential. We believe further progress in development of probes for FA monitoring in living objects could lead to breakthrough results and discoveries in the field of biochemistry of pathological conditions characterized by elevated levels of FA such as cancer and neurodegenerative disorders, providing new approaches for diagnostic and treatment.
